# PMERGE: Computational filtering of paralogous sequences from RAD‐seq data

**DOI:** 10.1002/ece3.4219

**Published:** 2018-06-11

**Authors:** Praveen Nadukkalam Ravindran, Paul Bentzen, Ian R. Bradbury, Robert G. Beiko

**Affiliations:** ^1^ Faculty of Computer Science Dalhousie University Halifax NS Canada; ^2^ Marine Gene Probe Laboratory Department of Biology Dalhousie University Halifax NS Canada; ^3^ Salmonids Section Science Branch Department of Fisheries and Oceans Canada St. John's NL Canada

**Keywords:** Atlantic salmon, paralogous sequence variants, RAD‐seq

## Abstract

Restriction‐site associated DNA sequencing (RAD‐seq) can identify and score thousands of genetic markers from a group of samples for population‐genetics studies. One challenge of de novo RAD‐seq analysis is to distinguish paralogous sequence variants (PSVs) from true single‐nucleotide polymorphisms (SNPs) associated with orthologous loci. In the absence of a reference genome, it is difficult to differentiate true SNPs from PSVs, and their impact on downstream analysis remains unclear. Here, we introduce a network‐based approach, PMERGE that connects fragments based on their DNA sequence similarity to identify probable PSVs. Applying our method to de novo RAD‐seq data from 150 Atlantic salmon (*Salmo salar*) samples collected from 15 locations across the Southern Newfoundland coast allowed the identification of 87% of total PSVs identified through alignment to the Atlantic salmon genome. Removal of these paralogs altered the inferred population structure, highlighting the potential impact of filtering in RAD‐seq analysis. PMERGE is also applied to a green crab (*Carcinus maenas*) data set consisting of 242 samples from 11 different locations and was successfully able to identify and remove the majority of paralogous loci (62%). The PMERGE software can be run as part of the widely used Stacks analysis package.

## 1 INTRODUCTION

Population‐genomic studies are surveying hundreds to thousands of markers to describe genome‐wide variation and make population‐genetic inferences (Hohenlohe et al. 2011). Restriction‐site associated DNA sequencing (RAD‐seq: Andrews, Good, Miller, Luikart, & Hohenlohe, 2016; Baird et al., 2008) uses next‐generation DNA sequencing methods and restriction enzymes to sample the genomes of nonmodel organisms. The genome is cut into fragments at restriction sites by restriction enzymes, and adapters are attached to the resulting DNA fragments for sequencing. Assembling these short DNA sequences into orthologous loci is a necessary step in the inference of true single‐nucleotide polymorphisms (SNPs), which serve as the basis for population‐level inference. Reference genomes can be used to map homologous sequences to their correct position in the genome, but studies of organisms that lack a reference genome depend on de novo locus assembly (e.g., Catchen, Hohenlohe, Bassham, Amores, & Cresko, 2013). This approach has limited capacity to differentiate paralogous sequence variants (PSV) from allelic SNPs (Waples, Seeb, & Seeb, 2016). PSVs can be erroneously merged together as a single locus, leading to the conflation of allelic variation with differences among closely related gene family members (Dou et al., 2012). Assembling paralogs as single loci increases false heterozygous genotype calls and can also confound genetic differentiation among individuals and populations, complicating genomic studies (Abadía‐Cardoso, Clemento, & Garza, 2011; David et al. 2007).

Stacks is a modular pipeline that processes RAD‐seq data to construct putative loci, either de novo from the short‐read DNA sequences (*ustacks*) or by alignment to a known reference genome (*pstacks*) in a set of individuals. Using *ustacks*, identical sequences from the raw RAD‐seq data of a given individual are assembled into putative alleles (primary stacks) if they satisfy a minimum sequence‐depth criterion *m*, and these primary stacks are then merged into putative loci in which all sequences have a nucleotide distance less than or equal to the chosen value of the *M* parameter. The remaining reads are merged into putative loci if they differ by *M *+* *2 nucleotides or less. The *ustacks* program performs de novo reconstruction of loci and identifies SNPs from the stacks separately for each individual sample in the population. The *cstacks* program builds the complete catalog of loci from all the individual samples in the entire population. The loci that are within a maximum nucleotide distance *n* are merged into one locus during the catalog building. Followed by the *cstacks* catalog building, the *sstacks* program identifies the matching catalog locus for each of the de novo locus in each individual. The *populations* program is used to generate population‐genetic statistics such as nucleotide diversity and *F*_ST_ (Holsinger & Weir, 2009; Weir & Cockerham, 1984).

**Figure 1:**
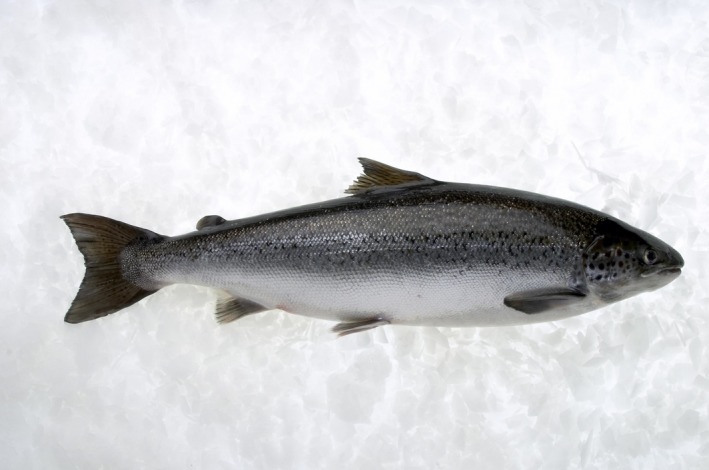
The Atlantic salmon (*Salmo salar*) is native to the northern Atlantic Ocean and north Atlantic rivers and also in North Pacific Ocean. The Atlantic salmon genome underwent a whole‐genome duplication (WGD) event 80 million years ago and is in the process of reverting to diploid state. Accessed from http://www.scienceimage.csiro.au/pages/about/on April‐2018

Duplication events at the gene, chromosome or genome level (Hurles, 2004) can create two or more paralogous DNA sequences from a single ancestral sequence and complicate genome assemblies and estimation of genetic variations (Davidson et al., 2010). The identification of loci and true SNPs from short sequences remains especially challenging for species with a duplicated genome because the duplicated sequences can be wrongly merged into a single locus, causing difficulty in identifying true allelic variations (Hohenlohe et al. 2011). A well‐known example is that of the salmonid fishes (Figure 1), which underwent a whole‐genome duplication event approximately 80 MYA (Lien et al., 2016; Macqueen & Johnston, 2014). In the absence of a reference genome, there are several methods available to filter paralogous loci from the genome data. Some approaches augment the genetic data with other information, such as linkage mapping based on pedigrees (Waples et al., 2016), and removing heterozygous SNPs from double‐haploid individuals (Limborg et al. 2016). Computational filtering approaches rely solely on the DNA sequence data, and can be performed either during assembly and genotyping (Dou et al., 2012; Eaton, 2014) or on the assembled data, for example, by retaining only those loci with the expected number of alleles and by retaining only those putative loci whose inferred genotype frequencies conform to Hardy–Weinberg equilibrium (HWE) expectations (Hardy 1908; Catchen et al., 2013; Hohenlohe, Amish, Catchen, Allendorf, & Luikart, 2011). For populations in HWE, the expected heterozygosity can never be more than 0.50 at any bi‐allelic locus. HDplot (McKinney, Waples, Seeb, & Seeb, 2017) uses read depths and excess heterozygosity to identify putative paralogs. HDplot works by plotting the relative proportion of heterozygotes in a population (*H*) and the deviation of allele‐specific reads of each locus from a 1:1 ratio (*D*). One more approach is haplotyping (Willis, Hollenbeck, Puritz, Gold, & Portnoy, 2017), which relies on the fact that closely linked SNPs can constitute haplotypes of which a diploid individual can have no more than two.

An important challenge in distinguishing paralogs is the choice of percent identity used to delineate loci; typically, a value of 98%–99% among reads is used (e.g., Catchen, Amores, Hohenlohe, Cresko, & Postlethwait, 2011; Lu et al., 2013). However, a stringent similarity threshold carries the risk of splitting divergent alleles into separate loci “over‐splitting” if the orthologs differ by an amount greater than the similarity threshold, whereas lower similarity thresholds can allow paralogous sequences to be incorrectly merged into one orthologous locus “under‐splitting” (Harvey et al., 2015; Rodríguez‐Ezpeleta et al., 2016). Stacks identifies the erroneously merged sequences and tries to break them into multiple loci using a deleveraging algorithm, which calculates a minimum‐spanning tree using the stacks from the locus as nodes and the distance between them as edge weights. The minimum edge weight for each node among all the edges connected to the node is identified, and the edges that are greater than the minimum edge weight are split. However, if the erroneous locus is formed from only 2 or 3 paralogous stacks, it will not be considered an over‐merged locus.

Given the potentially confounding effects of paralogous loci, new methods are needed to identify them and allow removal prior to the inference of population‐level statistics. Here we describe PMERGE, a new method that identifies candidate paralogs or duplicated loci in the catalog loci built by Stacks program. PMERGE works by building networks of catalog loci that share high levels of nucleotide similarity and flagging highly similar sequences as potential paralogs. Our approach is able to successfully identify the majority of paralogous loci generated from a RAD‐seq analysis of two species, first 150 sampled Atlantic salmon; and second 242 green crab (*Carcinus maenas*) samples. By embedding PMERGE in the analysis pipeline of the widely used Stacks software (Catchen et al., 2013), it is straightforward to apply it as an additional filter in population‐genomic studies using RAD‐seq data and can also be used in addition to other existing approaches.

## 2 METHODS

### 2.1 Identification of putative paralogs using PMERGE

**Figure 2:**
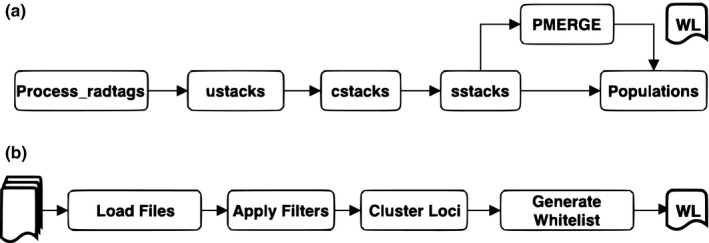
Use of PMERGE in the Stacks workflow to generate whitelisted (WL) sets of loci. (a) The modified Stacks pipeline with PMERGE invoked immediately after *sstacks* is used to search loci against the reference catalog. The *populations* program can use the whitelist file generated by the PMERGE module to include only those whitelisted loci. (b) The PMERGE module reads the catalog, tags, SNP, and match files, applies user‐defined filters and performs clustering to identify and eliminate paralogs. The retained loci are then written to a whitelist file

The PMERGE software (Figure 2) is run after *sstacks* and before *populations* to generate a “whitelist” of loci from the catalog based on population‐level filtering conditions and our new paralog‐detection method. The *populations* program then uses only the whitelisted loci to generate population‐genetic statistics. Aside from the paralog filter, PMERGE includes the following filters that are also used by the *populations* program: *percent samples limit per population* (r), which requires that a locus be present in at least the specified percentage of individuals in a population; *locus population limit* (p), the minimum number of populations in which a locus must be present; *minor allele frequency cutoff* (a), which sets a minimum threshold for the frequency of the minor allele (the second‐most‐frequent allele at a given locus) for each SNP in a locus; *maximum observed heterozygosity* (q) for each SNP in a locus; and *minimum stack depth* (m) at a given locus.

Paralogous sequences that have arisen from recent duplication events will exhibit high similarity with more than one region in the genome. The catalog built from the de novo loci formed from each individual gives us a pool of loci from all the individuals in the population, and our hypothesis is that highly similar groups within the pool of de novo loci have a high probability of being derived from multiple sites in the reference genome. PMERGE is applied to the catalog of loci and not to the de novo loci formed in each sample separately: the paralogs may be merged into a single locus in some but not all samples, allowing us to cluster them and identify based on similarity. PMERGE flags the polymorphic (heterozygous) catalog loci that are clustered with at least one other catalog locus.

To identify probable paralogs, we construct a graph or network where each node corresponds to a locus, which is represented by its consensus sequence. For efficiency, we represent all sequences of a given locus with a consensus sequence, which greatly simplifies the network; in practice, this reduction has minimal impact on the inference of paralogs. Using the consensus sequences can allow us to capture the cumulative mismatches of all the sequences in the locus. PMERGE uses a more‐lenient similarity threshold than *M* for clustering the catalog locus consensus sequences, which allows us to identify loci that are less similar than their constituent stacks but similar enough to be flagged as duplicates.

A cluster similarity threshold parameter *C* is set (default value 90%), and all pairs of loci whose representative sequences differ by no greater than *C*% are connected with an edge. Sets of loci that are connected by at least one path in the network define connected components; each of these components is interpreted as a putative set of paralogous sequences. These sequences can then be removed from the dataset prior to calculation of population parameters, or set aside for further analysis.

### 2.2 Validation of the proposed method

We validated the performance of PMERGE on an Atlantic salmon dataset (see Bradbury et al., 2015 and a green crab dataset (see Jeffery et al., 2017). For reference comparisons, polymorphic catalog loci consensus sequences were aligned to version ICSASG_v2 of the Atlantic salmon reference genome (Lien et al., 2016) and the Green crab reference (Hleap et al., in preparation) using BLASTN version 2.2.28 (Altschul, Gish, Miller, Myers, & Lipman, 1990) with a minimum of 90% sequence identity and a maximum E‐value of 1e‐20.

The Atlantic salmon dataset contained RAD‐seq data obtained from 150 individuals from 15 different locations along the south coast of Newfoundland, Canada. The dataset comprised samples with approximately 2,500,000–14,000,000 RAD‐tags per individual trimmed to 80 bp. The genomic DNA was digested using restriction enzyme *Sbf*I, and the resulting fragments were sequenced. Individually barcoded RAD samples were jointly sequenced on the Illumina GAIIx platform with single‐end 100‐bp chemistry. The Atlantic salmon genome underwent a whole‐genome duplication (WGD) event 80 million years ago and is in the process of reverting to diploid state (Allendorf & Thorgaard, 1984; Lien et al., 2016; Macqueen & Johnston, 2014; Ohno, Wolf, & Atkin, 1968). As such, salmon is a good choice for validation of our proposed method, as there is a reference genome available that allows us to verify the majority of our predictions by mapping loci back to the genome. We evaluated the number of paralogs identified by PMERGE with the Stacks de novo assembly similarity parameter *M* set to 2 and 4 in separate runs. The parameter “*C”* was varied from 90% to 50% with intervals of 10%, to compare the number of paralogs identified. Ilut, Nydam, and Hare (2014) recommended an *M* setting of 2 to reduce the merging of putative paralogs into one locus. De novo locus formation with *M *=* *4 was also carried out to demonstrate the effect of over‐merging in identifying the paralogs using the proposed approach. The catalog of loci is built using *cstacks* with maximum nucleotide distance allowed between catalog loci to merge *n = *1. The resulting catalog of assembled de novo loci was passed to our filtering software, with parameter settings *a *=* *0.05, *p *=* *12 and *r *=* *0.75.

The validation involved identifying the efficiency of PMERGE in correctly identifying and removing the paralogs in both data sets by aligning the identified paralogs to their corresponding reference genomes. First, all the polymorphic catalog loci were aligned to their reference genome and the loci with multiple hits to the reference genome flagged as candidate paralogs. Second, constituent alleles of each polymorphic catalog locus were aligned to the reference genome and alleles mapping to different regions in the reference genome were identified to flag the wrongly merged paralogs in the catalog. By comparing the polymorphic catalog loci flagged as paralogs by PMERGE with the candidate paralogs and the candidate PSVs the proportion of duplicated loci and PSVs identified by PMERGE was determined.

The impact of the parameter *M* on the results was also examined. The number of paralogous loci and PSVs identified by PMERGE was also compared with HDplot and filtering by deviations from H‐W expectations. The *populations* program in Stacks generates a VCF format output that was used as input for HDplot. The deviations from H‐W equilibrium were analyzed using VCFtools, which can use the VCF format output from Stacks. Loci that significantly deviate from HWE (*p* = 0.001) were flagged as paralogs. A combination of PMERGE and the two approaches was also performed to evaluate the possibility of improvement in the proportion of paralogs detected.

The error rates for different values of *C* were calculated by determining the number of paralogs (polymorphic loci with multiple hits to the reference genome) correctly identified in the clustered loci, and the number of nonparalogous sequences that were rejected in the analysis (false positives); the error rate for a given *C* was calculated as the ratio of the number of false positives to the total number of clustered loci. We used ROC (Receiver Operating Characteristic) curves to assess the performance of PMERGE in filtering paralogs. In a ROC curve, the true‐positive rates (in our case, detected paralogous loci) are plotted against the corresponding false‐positive rates (single‐locus alleles incorrectly classified by PMERGE as paralogous) for different values of a parameter, in our case *C*. The area under the resulting ROC curve (AUC) gives a measure of how well the method can distinguish between paralogous and nonparalogous loci.

One of the statistics calculated by the *populations* program in Stacks is pairwise *F*_ST_ values between all pairs of populations under study. As paralogs can affect population divergence estimates (Stuart et al. 2017), we compared the pairwise *F*_ST_ before and after paralog filtering by PMERGE on pairwise *F*_ST_. Dendrograms were generated from the pairwise *F*_ST_ distance matrices obtained with and without application of the proposed filter for *M* = 2 and *C* = 90%. The generated pairwise *F*_ST_ distance matrices were clustered using the “hclust” function in the R package “stats” (R Core Team 2015), which uses an agglomerative hierarchical clustering approach to construct relationships among different populations. In this analysis, the pairwise *F*_ST_ distance between populations was used as the distance metric, with clusters constructed based on the average‐linkage criterion, where the distance between two clusters of populations is defined as the average pairwise *F*_ST_ distance between each of their populations. The dendrograms created were mapped to the actual geographical locations using GenGIS (Parks et al., 2013). Differences in the topologies of the dendrograms created before and after PMERGE filtering were evaluated by calculating the Robinson‐Foulds distance (RF: Robinson & Foulds, 1981), as implemented in T‐REX (Boc, Diallo, & Makarenkov, 2012) and the rooted subtree prune‐and‐regraft (rSPR: Hein, Jiang, Wang, & Zhang, 1996; Whidden, Beiko, & Zeh, 2013) distances. The RF distance is the measure of number of bipartitions in one tree that are absent in the other tree. Migration of a single branch to a different part of the tree can affect many bipartitions, which inflates the RF distance and may overemphasize the distance between the two corresponding trees. An SPR operation cuts a subtree from the rest of the tree and reattaches it in a different location. The rSPR distance between two trees is equal to the minimum number of SPR operations required to reconcile two rooted trees, and is influenced less strongly by single branch migrations. RF and SPR therefore provide two contrasting views of tree similarity.

We also tested the ability of PMERGE to detect paralogs in a species that has no historical genome duplication. The green crab (*Carcinus maenas*) dataset consists of RAD‐seq data extracted from 242 individuals from 11 locations in eastern North America. Each library consists of 22 samples identified by variable length in‐line barcodes ranging from 5 to 9 bases. The libraries were sequenced on a HiSeq 2000 (Illumina) as 100 bp paired‐end sequences. The dataset comprises samples with approximately 3,000,000 RAD‐tags per individual trimmed to 80 bp. The RAD‐seq data from each individual sample were cleaned, demultiplexed and de novo assembled using the default Stacks parameters *M *=* *2 and *m = *3. The catalog of loci was built using *cstacks* with maximum nucleotide distance allowed between catalog loci to merge *n = *1. The resulting catalog of loci was then filtered using PMERGE with the parameter settings *a *=* *0.05, *p *=* *11 and *r *=* *0.75. In separate runs, the parameter “*C”* was varied from 90% to 40% with intervals of 10%, to compare the number of paralogs identified. Contrasting the Atlantic salmon genome, the absence of recent whole‐genome duplication in green crab lowers expectations of the prevalence of paralogs. The inclusion and comparison of both species allow the utility of PMERGE to resolve paralogs under two very different contexts to be evaluated.

## 3 RESULTS

We examined the effectiveness of PMERGE on data sets from two different species with distinct evolutionary histories. Both species have reference genomes available, which allow validation of paralogs predicted by PMERGE. First, we examined a set of RAD‐seq data from Atlantic salmon, which has a recent (80 MYA) whole‐genome duplication and consequently a large proportion of expected paralogs. The analyses included identifying the impact of filtering paralogs on the inferred population structure and random subsampling of loci to show that the differences in population structure after filtering the paralogs is not random. We also examined the removal of paralogs from a European green crab (*Carcinus maenas*) dataset, which was first used to study their population structure in Northwest Atlantic (Jeffery et al., 2017) and has no known historical genome duplication.

### 3.1 Atlantic salmon analysis

**Figure 3:**
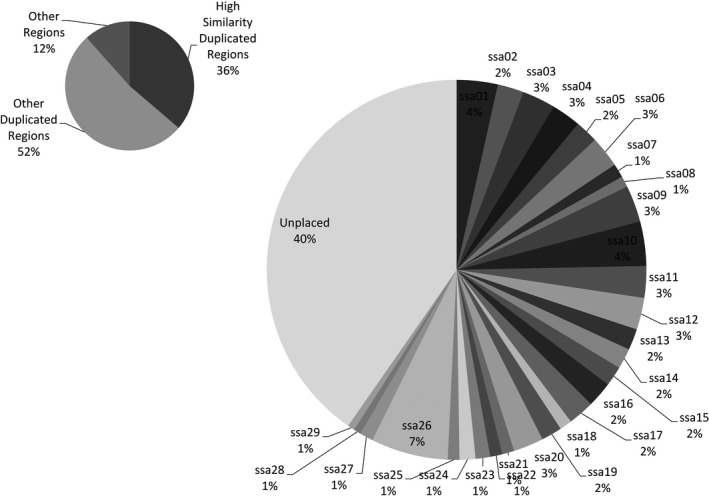
Distribution of putative paralogs (chromosome‐positioned) with respect to the chromosome regions. The putative paralogs are flagged from the catalog formed from Atlantic salmon dataset with de novo locus formation using *M* = 2 by aligning to the reference genome

For *M = *2, after applying the filters (see methods) 25,209 polymorphic catalog loci were retained and alignment of the locus consensus sequences to the Atlantic salmon reference genome using BLASTN revealed that 13,510 of these 25,209 were putative paralogs and mapped to multiple locations in the genome. In a similar manner, aligning the constituent allele sequences from the 13,510 catalog loci revealed that 4,852 loci (36%) had their allele sequences mapped to multiple regions in the reference genome. Out of the 13,510 putative paralogs, 5447 (40%) were unplaced and 8,063 were chromosome‐positioned (Figure 3). Approximately 36% of the 8,063 loci mapped to the homeologous blocks with high similarity (>90%) and 52% of the 8,063 loci are from the other homeologous blocks specified in Lien et al. (2016).

**Figure 4:**
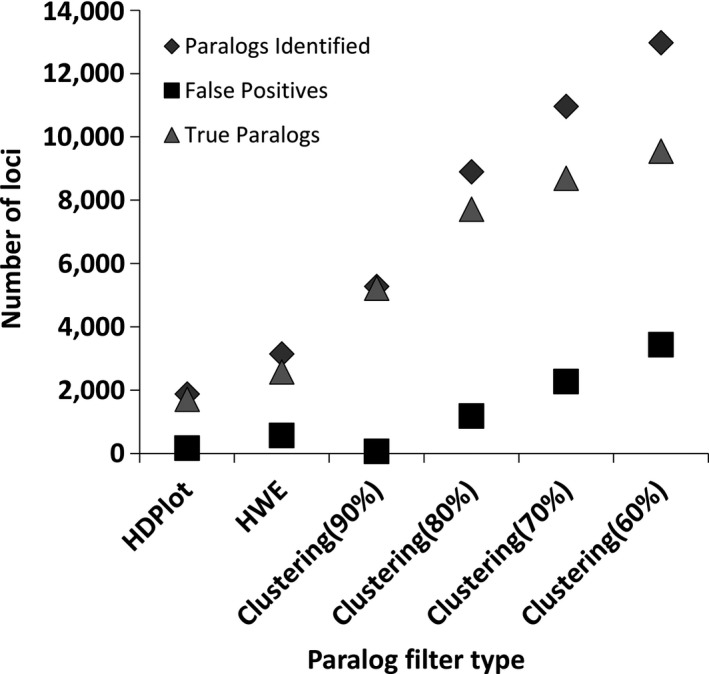
Comparing the effectiveness of the HDplot, HW approach and PMERGE for Atlantic salmon dataset with de novo locus formation using *M* = 2. Showing the number of putative paralogs identified, false positives and true paralogs identified by the HDplot, HW approach and PMERGE with different settings of cluster similarity *C*

From Figure 4, at *C *=* *90%, out of the 25,209 loci, 8,226 (32.63%) were clustered by PMERGE (i.e., potential paralogs) and 16,983 loci remained nonclustered. Of the 8,226 clustered loci, 8,214 (99.85%) mapped to multiple locations in the reference genome (Table S1a). Reducing *C* to 80% increased the number of loci clustered to 10,667 with 10,268 (96.26%) loci mapping to multiple locations in the genome. The cluster similarity threshold *C* was varied between 50% and 90% at intervals of 10% and the error rates recorded. Approximately 81% and 85% of the total putative paralogs were identified by *C = *70% and *C = *60% respectively. From *C = *90% to *C *=* *60%, the error rates varied from 0.01 to 0.10. At *C = *50%, all the 25,209 polymorphic loci were flagged as paralogs by PMERGE. On the other hand, using the HDplot approach, loci with the proportion of heterozygous individuals (*H*) > 0.6 and read‐ratio deviation (*D*) between −7 and 7 (Figure S1a) were flagged as paralogous. The HDplot approach identified 1996 loci as paralogs, out of which 167 loci uniquely mapped to the reference genome (false positives). The HWE filter identified 2,499 loci as paralogs, in which 566 loci were false positives. Approximately 36% and 45% of paralogs flagged by HDplot and deviations from HWE overlapped with paralogs identified by PMERGE (*C = *60%) respectively. PMERGE identified 1,938 loci with wrongly merged PSVs at *C *=* *90%, and the proportion increased as the similarity threshold *C* decreased. PMERGE identified a maximum of approximately 60% of the 4,852 merged PSVs at *C = *60%. HDplot identified 31% and deviation from HWE identified 30% of the loci with wrongly merged PSVs.

**Figure 5:**
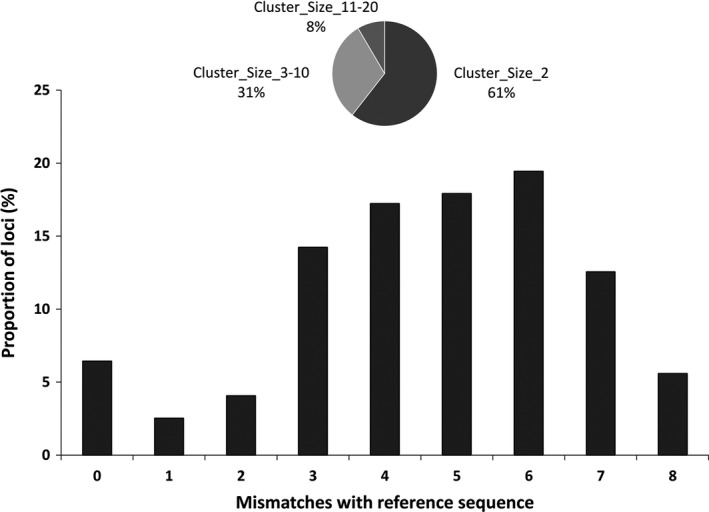
Distribution of filtered loci when *C *=* *90% that mapped exactly to two locations in the reference genome based on number of mismatches and on cluster size

When *C* = 90%, out of the 4,573 chromosome‐positioned loci 1211 (26.5%) were from high‐similarity duplicated regions and 2,649 (58%) were from other duplicated regions. Reducing *C* to 80%, out of the 5,667 chromosome‐positioned loci 1494 (26.4%) mapped to high‐similarity duplicated regions and 3340 (59%) mapped to other duplicated regions. At *C *=* *60%, a similar trend was observed, with 1,943 (28%) and 4,111 (59%) of the 6,981 chromosome‐positioned loci from high‐similarity duplicated regions and other duplicated regions respectively. From *C *=* *90% to *C *=* *60%, approximately 12%–16% of the loci were unplaced in the chromosome. Also, the distribution of the sequences flagged by PMERGE for different values of C with respect to the chromosome arm they are mapped indicates the filtered sequences is not from a specific region in the genome (Figure S2). Approximately 60% of the 8,226 loci that clustered at *C *=* *90% mapped to exactly two locations in the reference genome. Figure 5 shows the distribution of the loci mapped exactly to two locations with respect to the number of mismatches, including gaps. About 43% of the clustered loci that mapped exactly to two locations in the reference genome are part of clusters of size 2, and 71% of the loci with exactly two hits belong to clusters of size from 2 to 20. This illustrates the correlation between the size of the clusters and the number of matching locations in the genome.

**Figure 6:**
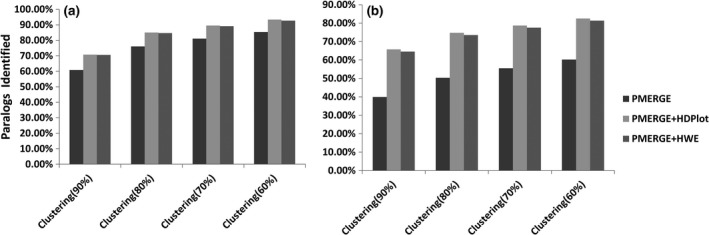
Paralogous loci identified using only PMERGE and in combination with HDPlot and HWE approaches using Atlantic salmon dataset with de novo locus formation using *M* = 2. (a) Putative paralogs idenitified. (b) Loci with merged paralogs detected

Combining PMERGE with HDPlot or deviation from HWE methods increased the proportion of paralogs and loci with wrongly merged PSVs identified (Figure 6). We observed an approximately 8% to 10% increase in the putative paralogs identified (Figure 6a) and a 22% to 26% increase in the loci with merged PSVs detected (Figure 6b). At *C *=* *60%, using only PMERGE we were able to identify 85% of the putative paralogs and 60% of loci with merged PSVs, whereas combining PMERGE with HDPlot or HWE approaches we were able to detect 93% of the putative paralogs and 81% of loci with merged PSVs.

When *M* = 4, there were 25,775 polymorphic loci were retained in the catalog for further analysis after applying filters. 12,473 loci out of the 25,775 mapped to multiple locations in the reference genome. Aligning the constituent alleles from the 12,473 loci revealed 4,316 loci (35%) had allele sequences that mapped to different regions in the genome. At *C *=* *90%, out of the 25,775 loci, 5254 (20.38%) were clustered by PMERGE (i.e., potential paralogs) and 16,983 loci remained nonclustered (Table S1b). Of the 5,254 clustered loci, 5239 (99.71%) mapped to multiple locations in the reference genome. Reducing *C* to 80% increased the number of loci clustered to 7,963 with 7,548 (94.79%) loci mapping to multiple locations in the genome. The error rates ranged from 0.01 to 0.13 for *C *=* *90% to *C *=* *60%, identifying 42% to 72% of the total paralogs respectively. Using the HDplot approach, loci with the proportion of heterozygous individuals (*H*) > 0.6 and read‐ratio deviation (*D*) between −7 and 7 (Figure S1b) were flagged as paralogous. The HDplot approach identified 1,880 loci as paralogs, out of which 124 loci uniquely mapped to the reference genome. The HWE filter identified 3,143 loci as paralogs, out of which 862 loci were false positives. Approximately 32% and 36% of paralogs flagged by HDplot and deviations from HWE overlapped with paralogs identified by PMERGE (*C = *60%) respectively. Approximately 22% and 32% of paralogs flagged by HDplot and deviations from HWE overlapped with paralogs identified by PMERGE (*C = *60%) respectively. PMERGE identified a maximum of approximately 50% of the 5,444 loci with merged PSVs at *C = *60%. Both HDplot and deviation from HWE identified 28% of the loci with merged PSVs.

**Figure 7:**
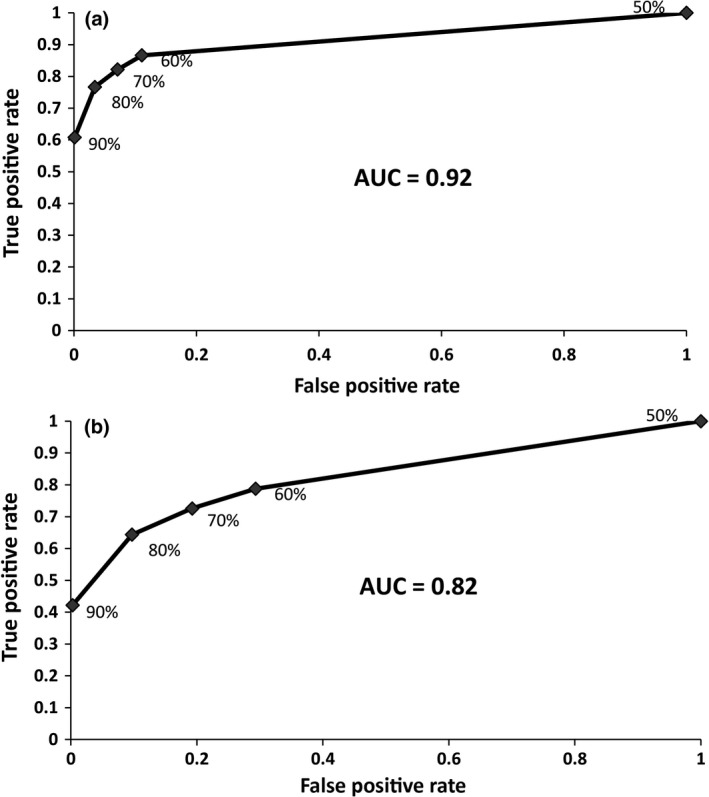
Identification of paralogous sequences by PMERGE. ROC curve generated using a. Atlantic salmon dataset with de novo locus formation using *M* = 2, (b) Atlantic salmon dataset with de novo locus formation using *M* = 4 The ROC curves are generated from the observed true positives (paralogs), true negatives (nonparalogs), false positives and false negatives. The percentage labels on the curves are the similarity thresholds *C* used. The area under the ROC curve demonstrates the accuracy of the proposed method

The ROC curve obtained using different values of *C* for *M *=* *2 (Figure 7a), the AUC was 0.92, which means PMERGE is good at separating paralogous loci from nonparalogous loci (Zweig & Campbell, 1993). Reducing the value of *C* below a certain limit leads to clustering of nonparalogous sequences (i.e., false positives). This is likely to happen because of short length of these sequences and the reduced amount of similarity required to cluster them. Also, the proposed method works based on only the similarity among the catalog of loci assembled from the set of samples, hence the number of paralogs identified is highly influenced by the proportion of similar loci available in the catalog. For *M *=* *4 (Figure 7b), the AUC reduced to 0.82, indicating the accuracy of PMERGE in separating paralogous loci from nonparalogous loci reduces as value of *M* increases.

### 3.2 Impact of paralog filtering on population structure

**Figure 8:**
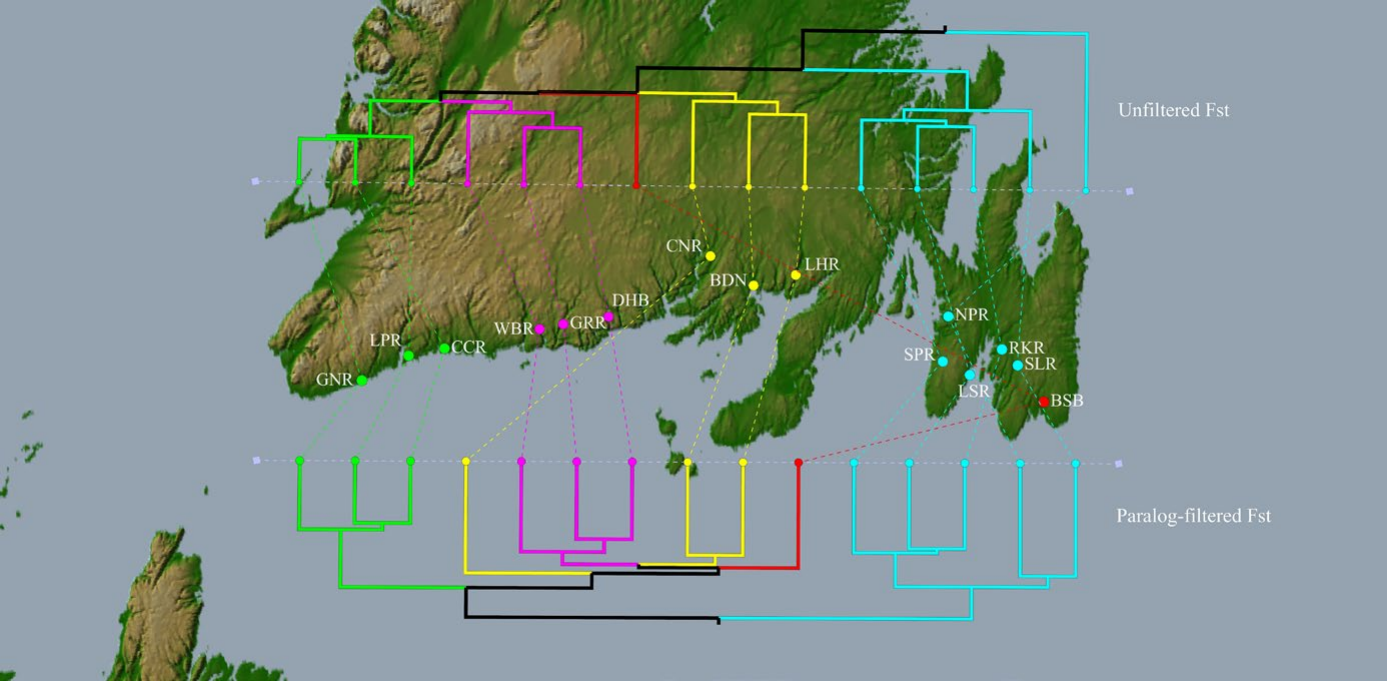
Dendrograms constructed from pairwise *F*_ST_ values between sites, before (top) and after (bottom) paralog filtering, with dendrogram leaves assigned to the sampled geographical locations along the Southern coast of Newfoundland. The C parameter was set to 90% and there were 40,618 loci before paralog filtering and 32,392 loci after paralog filtering

Pairwise *F*_ST_ values after applying the paralog filtering generally increased between the populations. For the site “NPR” the pairwise *F*_ST_ values with other sites generally decreased after applying the paralog filtering, except with the sites “BSB,” “LSR,” “RKR,” and “SPR” where the pairwise *F*_ST_ values increased. While the percentage difference in the pairwise *F*_ST_ values after applying PMERGE filtering was as low as 0.96% between “NPR” and “LSR,” it was as high as 28.95% between “SLR” and “BSB.” Dendrograms obtained from the pairwise *F*_ST_ values generated between all pairs of populations under study before and after applying the PMERGE filter differed in topology. Figure 8 shows variations in subpopulation structures between the unfiltered and PMERGE‐filtered trees: one notable pattern is the increased genetic differentiation between the east and west coast populations with the paralog‐filtered data. In the paralog‐filtered dendrogram, there are two major clusters separating the east and west coast populations, and the five populations “SPR,” “SLR,” “RKR,” “NPR,” and “LSR” from the Avalon Peninsula in the east are grouped into one cluster. However, with the unfiltered data, one of those five east coast populations “NPR” is an outlier in the generated dendrogram. The clustering of “NPR” with the rest of the populations from the Avalon Peninsula is a result of the differences in their pairwise *F*_ST_ values after applying the PMERGE paralog filter, as opposed to using *F*_ST_ values obtained from the unfiltered data.

**Figure 9:**
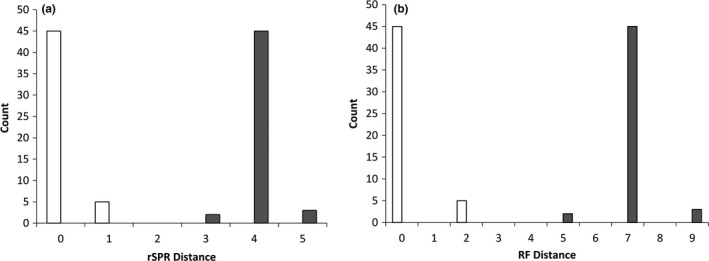
Distribution of rSPR (a) and RF (b) distances between trees constructed from randomly subsampled loci, and trees obtained from pairwise *F*_ST_ values before (white bars) and after (gray bars) PMERGE filtering. The RF distance is the measure of number of bipartitions in one tree that are absent in the other tree and rSPR distance is minimum number of SPR operations required to reconcile two rooted trees

Robinson‐Foulds distance and rSPR distances (Figure 9) calculated to compare the topology of the dendrograms were 10 and 4, respectively, indicating differences between the dendrograms. As the PMERGE‐filtered dendrogram was based on fewer loci than the unfiltered tree, we assessed the impact of choosing random subsamples of 32,392 loci from the unfiltered tree. Fifty replicate trees based on random subsamples of loci were constructed. If the effect of paralog filtering is greater than that of random subsampling, we expect that the paralog‐filtered tree should differ more from the reference tree than do the dendrograms obtained from random subsamples. The rSPR distances between the unfiltered *F*_ST_ dendrogram and the dendrograms constructed from randomly subsampled loci were between 0 and 1, whereas the corresponding distances for the filtered *F*_ST_ dendrogram ranged between 3 and 5 (Figure 9a). The RF distances showed a similar trend but with distance values of 0–2 for unfiltered *F*_ST_ dendrogram and 5–9 for filtered *F*_ST_ dendrogram (Figure 9b).

### 3.3 Green crab analysis

**Figure 10:**
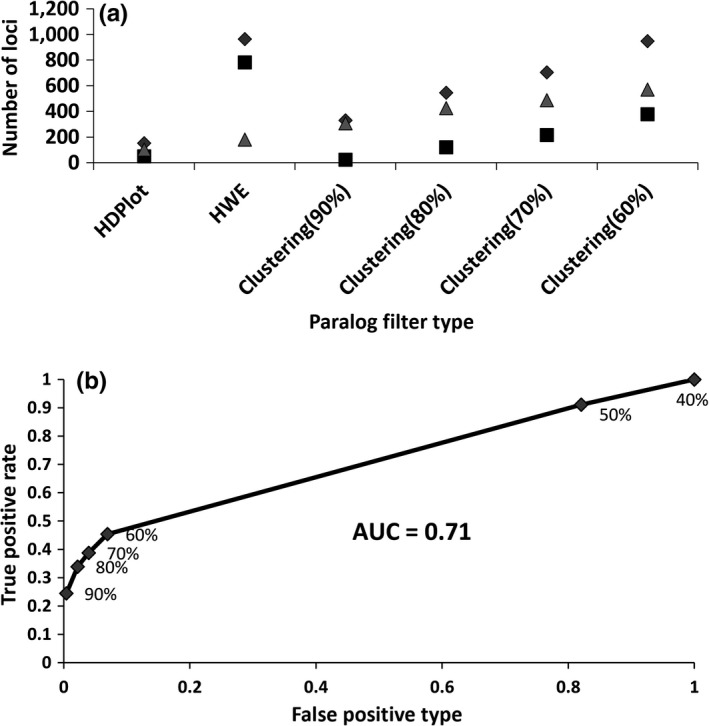
Paralog filtering in green crab dataset using HDplot, HW approach and PMERGE. (a) Comparing effectiveness of PMERGE and other methods. (b) ROC curve generated using the observed true positives (paralogs), true negatives (nonparalogs), false positives and false negatives

In contrast with the Atlantic salmon genome, the green crab has no evidence of ancestral genome duplication; consequently, far fewer paralogs are expected. Assembling these RAD‐tags using *ustacks* yielded approximately 25,000 loci per individual. The complete catalog contained 156,272 unique loci, which decreased to 12,435 by applying the locus filters as described in the Methods section. Of these 12,435 loci, 6695 were polymorphic and alignment to the green crab reference (Hleap et al., in preparation) genome using BLASTN revealed that 913 of these 6,695 mapped to multiple locations in the genome (putative paralogs) and 360 loci with alleles that mapped to different locations in the genome (loci with merged PSVs). We have also compared the effectiveness of PMERGE with other approaches and performed ROC curve analysis (Figure 10). At *C *=* *90%, out of the 12,435 loci, 330 (32.63%) were clustered by PMERGE (i.e., potential paralogs) and 12,105 loci remained nonclustered (Table S1c). Of the 330 clustered loci, 307 (93%) mapped to multiple locations in the reference genome (Figure 10a). Reducing *C* to 80% increased the number of loci clustered to 546 with 426 (78%) loci mapping to multiple locations in the genome. PMERGE identified a maximum of 62% of total paralogs and 37% of total loci with merged PSVs at *C *=* *60%. The error rates ranged from 0.07 to 0.40 for *C *=* *90% to *C *=* *60%. The HDplot approach flagged 153 loci with proportion of heterozygous individuals (*H*) > 0.6 and read‐ratio deviation (*D*) between −10 and 10 (Figure S1c) as paralogous. 50 out of the 153 loci mapped uniquely to the reference genome and 40 loci were PSVs (11% of the 360 loci). Using the HWE method, 963 loci were flagged as paralogs in which, 782 loci were false positives and 75 were merged PSVs (21% of the 360 loci). The AUC for the ROC curve obtained using different values of *C* was 0.71 (Figure 10b).

## 4 DISCUSSION

The splitting of paralogous loci depends on the choice of maximum nucleotide distance parameter (*M* in the Stacks software); as *M* increases, paralogous loci are merged together (Harvey et al., 2015; Rodríguez‐Ezpeleta et al., 2016). The putative paralogs flagged by PMERGE are the catalog loci with high sequence similarity. PMERGE identifies the wrongly merged PSVs by considering the entire catalog of loci constructed from all samples, rather than focusing on one sample at a time. If paralogs are merged into a single locus in one or more samples and not in others, the resulting pattern is used by PMERGE to properly subdivide loci.

Using the Atlantic salmon dataset we were able to assess the extent to which paralogs identified by PMERGE mapped to two or more genomic regions. When *M* = 2, 36% of the putative paralogs that mapped to chromosomes were situated in homeologous blocks with high similarity (>90%), and an additional 52% mapped to other homeologous blocks specified in Lien et al. (2016). Mapping the paralogs flagged by PMERGE for different values of *C* revealed that approximately 26% to 28% of the chromosome‐positioned loci were from the high‐similarity regions. Around 58% to 59% of the loci mapped to the less similar duplicated regions. As the sequences used in the analysis are as short as 80 bp, we see high similarity among them even though they are from less similarity duplicated regions and wrongly merged into a locus.

Comparing the paralogs identified by HDplot, deviations from HWE and PMERGE with different values of *C*, it is evident that PMERGE identifies more number of paralogs and merged PSVs than other two methods. The HDplot and deviations from HWE focus on identifying the merged PSVs by analyzing individual polymorphic loci, whereas PMERGE identifies paralogous loci in the catalog using their similarity. While HDPlot and HWE tests are applied to the VCF format output generated by the *populations* program in Stacks, PMERGE is applied to the catalog loci before the *populations* program even processes them. Most of the PMERGE flagged loci with merged PSVs were unique to PMERGE. Combining PMERGE with the other two approaches increased the proportion of paralogs detected. Approximately, 7% of the paralogous loci and 19% of the wrongly merged PSVs were not detected by any of the three approaches. As PMERGE cannot subdivide loci that are merged across all samples, the best use case, explored above and worthy of further development, is to combine the PMERGE approach with other methods such as HDplot which can examine distributional patterns within loci from even a single sample.

By observing the number of loci clustered for different values of *C*, we can identify an optimal cutoff value for this parameter, depending on the species and dataset. The accuracy of PMERGE analyzed using AUC obtained from ROC curves suggests that PMERGE can best perform when the species has more duplicated regions and the M value used in Stacks is low. The AUC obtained for the Atlantic salmon data at *M *=* *2 was 0.92 and for *M *=* *4 it was 0.82, whereas we obtained an AUC of 0.71 for the green crab dataset. Unlike the Atlantic salmon, the green crab does not have large proportions of highly similar regions in the genome. Hence the accuracy of PMERGE in separating paralogous loci from nonparalogous loci is less than salmon data. For large values of *M* and species with less duplicates setting the similarity threshold *C* in PMERGE to high values (more than 80%) and as low as 60% for smaller values of *M* and species with genome duplication.

Applying PMERGE with a *C* value as high as 90% eliminated at least 61% of paralogous loci and 40% of the loci with wrongly merged paralogs. The resulting population structure is more consistent with the previous study by Bradbury et al. (2015) involving microsatellites, SNP arrays and RAD‐seq data from southern Newfoundland, that also showed strong evidence of subdivision of salmon populations into eastern and western groups. In the RAD‐seq data used for their analysis, the PSVs were eliminated by removal of SNPs with three or more alleles as well as SNPs that mapped to multiple locations in the reference genome (Davidson et al., 2010). As expected, the populations were clustered into two large east–west groups. Analysing the dendrogram obtained without applying PMERGE filtering, the “NPR” population was unusually distinct, contradicting the results obtained in previous studies. The RF and rSPR distance comparisons between the paralog‐filtered dendrogram, the dendrogram obtained without applying paralog filtering and the dendrograms obtained from the random subsample showed that paralog filtering applied using PMERGE has a significant nonrandom effect on the topology of the pairwise *F*_ST_ dendrogram.

## 5 CONCLUSIONS

We have demonstrated the effectiveness of PMERGE in filtering paralogous loci from two species with different genome structures. Depending on the species under study and the expected proportion of paralogs, different values of *C* may be examined for an optimal value based on the proportion of detected paralogs. Also, for nonmodel species, we will not know the expected proportion of paralogs and in that case, the best option will be to set high values for *C*. The results from the Atlantic salmon and green crab datasets show that we can detect large number of paralogs even with high values of *C* using PMERGE.

## 6 SOFTWARE AVAILABILITY

Considering the large volume of data sets used for population genetics studies using short reads, the implementation of this approach focused on making it less computationally demanding. The implementation is done in C++ on a UNIX/LINUX platform. The PMERGE software, which includes parallel processing options using OpenMP API (Leonardo & Ramesh, 1998), is available at github.com/beiko‐lab/PMERGE.

## 7 SUMMARY

PMERGE uses a network‐based approach to identify probable paralogous site variants based on the similarity of inferred loci. We evaluated the proposed method using RAD‐seq data from 150 Atlantic salmon samples collected from 15 locations across the Southern Newfoundland coast and 242 green crab samples from 11 different locations in eastern North America. The analysis with green crab shows that PMERGE can be used as an effective paralog filter in other species as well. Although there is no globally optimal value for *C*, analysis of the Atlantic salmon and green crab datasets yielded a substantial reduction of paralogs even if we choose values of *C* as high as 80%. The extent of genome duplication in the species and the proportion of duplicated genomic sequences in the datasets play an important role in the choice of *C*. Also, the divergence of the duplicated genomic regions can affect the clusters formed for a given *C*. PMERGE can identify paralogs without aligning the sequences to a reference genome and so can be applied to species with no or partial reference genome. PMERGE is fast and easy to integrate in the RAD‐seq data processing and analysis. PMERGE can also be used along with other existing paralog detection methods for improved efficiency. The PMERGE package implements the proposed approach to identify paralogs and provides other locus filtering options available in the *populations* program in Stacks.

## CONFLICT OF INTEREST

None declared.

## AUTHORS CONTRIBUTION

All authors aided in the conception of the work. PNR implemented the software and conducted analyses. All authors contributed to writing, revising, and approving the final draft of the manuscript.

## DATA ACCESSIBILITY

Atlantic salmon DNA sequences: All raw RAD‐seq reads data can be accessed at NCBI SRA. Bioproject # PRJNA291587, Biosample #s SAMN04053806‐SAMN04053964.

Green crab DNA sequences: Raw RAD‐seq reads are available from the NCBI Sequence Read Archive BioProject PRJNA377723 at https://www.ncbi.nlm.nih.gov/Traces/study/?acc=SRP102198.
