# Supplementary material for: PMERGE: Computational filtering of paralogous sequences from RAD‐seq data
Source: Ecol Evol. 2018 Jun 11;8(14):7002–13. doi: 10.1002/ece3.4219 (PMC6065343; doi:10.1002/ece3.4219)
Supplement: Supplementary file 1 [file ECE3-8-7002-s001.docx]

**Figure 1**. H and D plot obtained for A.) Atlantic salmon data set with *de novo* locus formation using M=2, B.) Atlantic salmon data set with *de novo* locus formation using M=4 and C.) Green crab data set with *de novo* locus formation using M=2.

A.


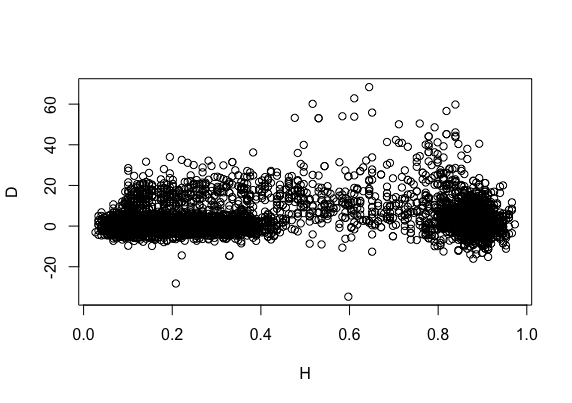


B.


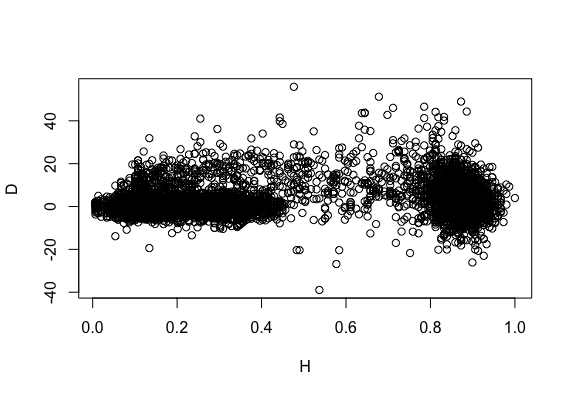


C.


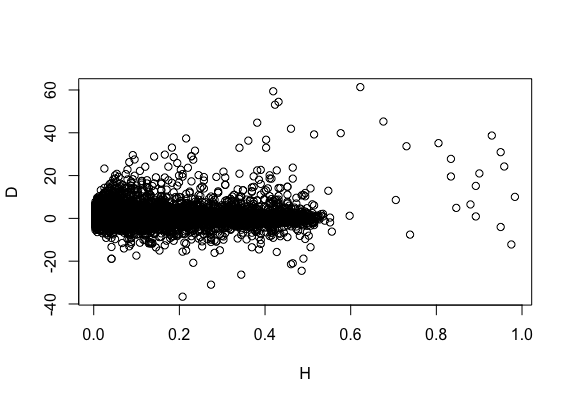


**Figure 2.** Distribution of the sequences flagged by PMERGE using Atlantic salmon data set with *de novo* locus formation using M=2,for different values of C with respect to the chromosome arm they are mapped.


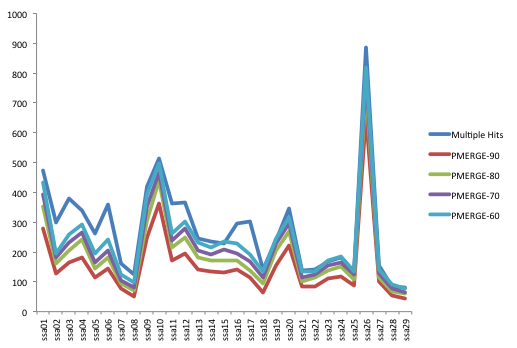


**Table 1.** Paralogs detected using HDPlot, deviations from HWE and PMERGE (different values of C) using A.) Atlantic salmon data set with *de novo* locus formation using M=2, B.) Atlantic salmon data set with *de novo* locus formation using M=4 and C.) Green crab data set with *de novo* locus formation using M=2.

A.

|  |  |  |  |  |  |
| --- | --- | --- | --- | --- | --- |
| **Method** | **Paralogs Flagged** | **True Positives** | **False Positives** | **% True Paralogs** | **Error rate** |
| **HWE** | 2225 | 1799 | 426 | 0.81 | 0.19 |
| **HDPlot** | 1805 | 1683 | 122 | 0.93 | 0.07 |
| **PMERGE-90** | 8226 | 8214 | 12 | 1.00 | 0.00 |
| **PMERGE-80** | 10667 | 10268 | 399 | 0.96 | 0.04 |
| **PMERGE-70** | 11786 | 10953 | 833 | 0.93 | 0.07 |
| **PMERGE-60** | 12829 | 11532 | 1297 | 0.90 | 0.10 |

B.

|  |  |  |  |  |  |
| --- | --- | --- | --- | --- | --- |
| **Method** | **Paralogs Flagged** | **Multiple hits in genome** | **False Positives** | **% True Paralogs** | **Error rate** |
| **HWE** | 3143 | 2281 | 862 | 0.73 | 0.27 |
| **HDPlot** | 1880 | 1756 | 124 | 0.93 | 0.07 |
| **PMERGE-90** | 5254 | 5239 | 36 | 1.00 | 0.00 |
| **PMERGE-80** | 7963 | 7548 | 1284 | 0.95 | 0.05 |
| **PMERGE-70** | 9231 | 8333 | 2544 | 0.90 | 0.10 |
| **PMERGE-60** | 10360 | 8979 | 3862 | 0.87 | 0.13 |

C.

| **Method** | **Paralogs Flagged** | **True Positives** | **False Positives** | **% True Paralogs** | **Error rate** |
| --- | --- | --- | --- | --- | --- |
| **HWE** | 963 | 181 | 782 | 0.19 | 0.81 |
| **HDPlot** | 153 | 103 | 50 | 0.67 | 0.33 |
| **PMERGE-90** | 330 | 307 | 23 | 0.93 | 0.07 |
| **PMERGE-80** | 546 | 426 | 120 | 0.78 | 0.22 |
| **PMERGE-70** | 703 | 487 | 216 | 0.69 | 0.31 |
| **PMERGE-60** | 947 | 570 | 377 | 0.60 | 0.40 |
| **PMERGE-50** | 5607 | 836 | 4771 | 0.15 | 0.85 |
